# TRPC6 is ubiquitously present in lymphatic tissues: A study using samples from body donors

**DOI:** 10.3892/mi.2024.186

**Published:** 2024-08-02

**Authors:** Felix Daum, Fidelis Flockerzi, Alessandro Bozzato, Bernhard Schick, Thomas Tschernig

**Affiliations:** 1 Institute of Anatomy and Cell Biology, Faculty of Medicine, Saarland University, D-66421 Homburg/Saar, Germany; 2 Institute of Pathology, Faculty of Medicine, Saarland University, D-66421 Homburg/Saar, Germany; 3 Department of Otorhinolaryngology, Faculty of Medicine, Saarland University, D-66421 Homburg/Saar, Germany

**Keywords:** TRPC6, calcium signaling, cation channel, lymphatic tissue, immunohistochemistry, immunology, leucocyte migration, lymphoma, lymph nodes, spleen, palatine tonsil, ileum, vermiform appendix, thymus

## Abstract

Transient receptor potential canonical channel 6 (TRPC6) is a non-selective cation channel that is activated by diacylglycerol. It belongs to the TRP superfamily, is expressed in numerous tissues and has been shown to be associated with diseases, such as focal segmental glomerulosclerosis, idiopathic pulmonary arterial hypertension and cardiac hypertrophy. The investigation of the channel in human lymphoid tissues has thus far been limited to mRNA analysis or the western blotting of isolated lymphoid cell lines. The present study aimed to detect the channel in human lymphoid tissue using immunohistochemistry. For this purpose, lymphatic tissues were obtained from body donors. The lymphatic organs analyzed included the lymph nodes, spleen, palatine tonsil, gut-associated lymphoid tissues (ileum and vermiform appendix) and thymus. A total of 102 samples were obtained and processed for hematoxylin and eosin (H&E) staining. The H&E staining method was employed to identify five samples with good morphology. In total, three samples of the palatine tonsil of patients were included. Immunostaining was carried out using a knockout-validated anti-TRPC6 antibody. As shown by the results, using immunohistochemical staining, the presence of TRPC6 was confirmed in all the analyzed lymphatic tissue samples. Lymphocytes in lymph nodes, spleen, palatine tonsil, thymus, and gut-associated lymphatic tissues in ileum and vermiform appendix exhibited a positive staining signal. The follicle-associated epithelium of the palatine tonsil, ileum and appendix also demonstrated staining. Vessels of the lymphatic organs, particularly the trabecular arteries of the spleen, the submucosal vessels of the appendix and ileum, as well as the high endothelial venules in the palatine tonsils and lymphatic vessels of the lymph nodes expressed TRPC6 protein. TRPC6 in follicles may be involved in the immune response. TRPC6 in high endothelial venules suggests a role in leukocyte migration. The role of TRPC6 and other channels of the TRP family in lymphatic organs warrant further investigations to elucidate whether TRP channels are a pharmacological target.

## Introduction

Transient receptor potential (TRP) channels constitute a family of unselective cation channels, the majority of which are permeable to Ca^2+^ (1). Alterations in the cytosolic Ca^2+^ concentration play a pivotal role in fundamental cellular processes, including the release of transmitters, cell proliferation, gene transcription and cell death (2). In lymphocytes, the TRP channels play an essential role in the calcium-mediated inflammatory response of the immune system (3).

The TRP channels were first described in 1969 in the fruit fly *Drosophila melanogaster* and have since been divided into subfamilies (4,5). The subfamily with the greatest homology to the channel discovered in the fruit fly is designated as TRP canonical (TRPC, meaning ‘standard’). The family TRPV vanilloid (TRPV) is named after the first member of this family, which is designated TRPV1 (formerly known as vanilloid receptor 1). The family with homology to melastin-1 is designated as TRPM. The families TRPP and TRPML are named for their inclusion of polycystin and mucolipin, respectively. The TRPA family is named for its abundance of ankyrin repeats (5). Finally, the TRPN family (NOMP, no mechanopotential) is worthy of mention. Genomic analysis indicates that this channel is not expressed in mammals (6).

Seven TRPC subunits have been identified in mammals, with TRPC2 being pseudogenized in humans and thus, not expressed (7). The family can be further subdivided based on homology in the amino acid sequence: TRPC1, TRPC2 and TRPC3/6/7(8). All TRPC channels are composed of six transmembrane helices, which then form a TRPC monomer (9). Four of these monomers then form a TRPC homotetramer, which represents the functional cation channel. However, the formation of heterotetramers is also possible, although these are limited to the individual subdivisions, such as TRPC3/6/7(10).

All TRPC channels, including TRPC6, require phospholipase C (PLC) for activation (11,12). The PLC pathway is most likely initiated by a G_q/11_-coupled receptor, which hydrolyses phosphatidylinositol 4,5-bisphosphate at the plasma membrane to inositol 1,4,5-trisphosphate (IP_3_) and diacylglycerol (DAG). IP_3_ can then activate IP_3_ receptors on the endoplasmic reticulum (13). DAG has been demonstrated to directly activate the TRPC6 channel (14). This form of activation and the resulting influx of Ca^2+^ into the cell is referred to as receptor-operated Ca^2+^ entry (15). However, it appears that DAG is not the sole means of channel activation (16). Another potential avenue is the store-operated Ca^2+^ entry, whereby the TRPC and Orai channels form a unified entity that becomes active in response to a decline in intracellular Ca^2+^ levels within intracellular stores (17).

A mutation of the TRPC6 gene is a cause of a genetically inherited form of focal and segmental glomerulosclerosis. The pathogenic mechanism is considered to be a gain-of-function mutation and an associated calcium overload (18,19). It is also known that the calcium homeostasis influenced by TRPC6 affects immunological processes. A single nucleotide polymorphism on the TRPC6 gene has been demonstrated to protect against the development of neuropsychiatric manifestations associated with systemic lupus erythematosus (20). Additionally, there is evidence to suggest that the reduced expression of TRPC6 inhibits the proliferation of human Burkitt lymphoma cells (21). It was hypothesized that the increase in intracellular calcium levels, which is mediated by TRPC6, stimulates cell proliferation (21). In addition to lymphoma cells, a connection has been established between TRPC6 and tumor entities, such as breast, cervical, gastric, esophageal cancer and gliomas. For all these cancer types, an elevated expression of the channel has been documented (22-26).

In experiments conducted on TRPC6 knockout mice, a reduction in allergic responses and IgE levels has been observed. The examined T-helper cells (Th2) exhibited lower levels of interleukin (IL)-5 and IL-3 secretion in comparison to the wild-type cells (27). It has been demonstrated that TRPC6 represents the principal channel regulating leukocyte migration (28). This finding builds on the understanding that an increase in intracellular free Ca^2+^ plays a pivotal role in this process, a concept which had already been established (29). Furthermore, the application of western blotting and calcium imaging techniques enabled the demonstration that septic peripheral blood T-lymphocytes from rats exhibited augmented expression of TRPC6(3). This expression is associated with both the activation of the T-cells and their release of cytokines (30).

The current literature indicates that the TRPC6 channel may be ubiquitously expressed in human lymphatic tissues, potentially involved in a range of functions. The TRPC6 channel has been identified in human lymphatic tissues, particularly the spleen, using northern blotting (31). Furthermore, the channel was previously identified using reverse transcriptase-polymerase chain reaction (RT-PCR) and western blotting in human peripheral blood T-lymphocytes and Jurkat T-lymphocytes (32). RT-PCR demonstrated TRPC6 expression in murine tissues, with a differential expression between B- and T-lymphocytes. The expression of the TRPC6 gene was significantly higher in B-lymphocytes than in T-lymphocytes (33). Additionally, differences in gene expression were observed between lymphocytes from different lymphoid organs. In these experiments, splenocytes exhibited a more robust expression than lymphocytes from lymph nodes or the thymus. However, the thymus, spleen, and lymph nodes exhibited a positive detection of TRPC6 mRNA (33).

A direct examination of the protein and an overview of the TRPC6 channel in human lymphatic tissues have not yet been conducted, at least to the best of our knowledge. Consequently, the present study aimed to investigate human lymphatic tissues using immunohistochemistry (IHC) to gain a deeper understanding of the potential immunological function of the channel.

## Materials and methods

The objective of the applied methods was to identify suitable human tissue for subsequent IHC staining to enable the investigation of the TRPC6 channel. This was achieved by obtaining tissue samples from lymphatic organs, including lymph nodes, spleen, vermiform appendix, ileum and thymus from body donors. Samples of the palatine tonsil were obtained during the course of planned tonsillectomies. The samples were initially embedded and cut with a microtome before undergoing hematoxylin and eosin (H&E) staining. Using the evaluation of the H&E-stained slides, five samples of each lymphatic organ from body donors with good morphology were selected and processed for IHC. In total, three samples of palatine tonsils from patients were processed for IHC.

### Specimens

A total of three tonsil samples were provided by the Department of Otorhinolaryngology (Saarland University, Campus Homburg/Saar, Germany). All other tissue samples were obtained from body donors at the Institute of Anatomy (Saarland University, Campus Homburg/Saar, Germany). Body donors (n=35) were embalmed with a combination of nitric pickling salt, ethanol and a low formaldehyde concentration or with a high formaldehyde concentration (all solutions from Otto Fischar GmbH & Co. KG). The first method was developed by Janczyk *et al* (34). The fixation of the corpses occurred within a period of <72 h post-mortem. A total of 102 samples were obtained from the body donors. A second collection of samples was taken from the ileum, vermiform appendix and thymus due to the difficulty in accessing these tissues. The resulting samples are presented in Table I. An overview of the associated body donors of the specimens subjected to IHC is presented in Table II. The fixation method, as well as the immediate cause of death, age at death/at surgery, and sex of the donors are listed in Table II.

All abdominal organs (ileum, vermiform appendix and spleen) were excised via median laparotomy. Thymus samples were obtained via median sternotomy from retrosternal adipose tissue. Lymph nodes were harvested through an incision at the level of the common femoral artery. A subsequent examination revealed that retrosternal adipose tissue also contained lymph nodes. Following excision, the tissue samples were immersed in 4% formaldehyde (overnight, 4˚C) and subjected to paraffin embedding. The embedded tissues were then sectioned into 7-µm-thick slices using a microtome (Leica RM 2026, Leica Microsystems GmbH). H&E staining was conducted in accordance with standard protocols, and IHC was performed as previously described (35), with additional details provided below.Klicken oder tippen Sie hier, um Text einzugeben.

### IHC

IHC staining was conducted using a primary anti-TRPC6 antibody (cat. no. ACC-017; Alomone Labs), which has been validated for specificity through knockout validation and peptide incubations conducted in previous studies (36,37). Prior to the application of the primary antibody, heat-induced epitope retrieval (HIER) and blocking were performed. For HIER, the sections were incubated in a 90˚C citrate buffer solution (Abcam, Cambridge, UK) for 60 min. For blocking, the sections were washed in phosphate-buffered saline (Carl Roth GmbH & Co. KG) and incubated with normal goat serum (cat. no. 01-6201; Invitrogen AG; Thermo Fisher Scientific, Inc.). The sections were incubated with the primary antibody at a concentration of 1:50 for 12 h, with negative and positive controls being employed at each staining cycle. The negative controls were incubated with rabbit serum (cat. no. PLN5001; Life Technologies; Thermo Fisher Scientific, Inc.) in place of the primary antibody. The positive controls were of a section of cardiac muscle of previously conducted studies by Jacobs *et al* (36), as this tissue has already been shown to express the TRPC6 protein by IHC. In order to achieve the best possible comparability of the individual staining runs, we used the same sample of cardiac muscle from Jacobs *et al* each time. This also allowed for a direct comparison with the peptide incubations studied by Jacobs *et al* (36). A secondary antibody, horseradish peroxidase (HRP)-conjugated (cat. no. A10547; Invitrogen AG; Thermo Fisher Scientific Inc.), was used at a dilution of 1:500. The sections were incubated for 60 min at room temperature. The chromogen utilized was diaminobenzidine (cat. no. SK-4103; Vector Laboratories, Inc.), which was incubated with the sections for 10 min at room temperature. The chromogen exhibited a brown coloration at the sites of antibody binding, resulting from the reaction with the HRP of the secondary antibody. The sections were subsequently counterstained with hematoxylin (Carl Roth GmbH & Co. KG, Germany) at room temperature for <1 sec to achieve a minimal counterstain that did not obscure the IHC staining.

### Analysis

The sections were examined using a light microscope with a camera (MikroCam SP 5.1; Bresser, GmbH) to assess the degree of browning. The sections were classified into three categories based on the intensity of the brown coloration: Strong, weak and negative. The negative sections exhibited only blue hematoxylin counterstaining.

## Results

The results of the analysis were divided into the various lymphatic organs that were evaluated. For each organ, structures were defined and analyzed individually.

### Lymph nodes

**Figure 1:**
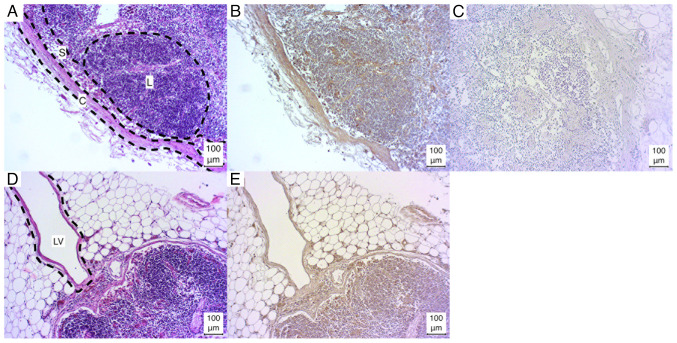
Microphotographs of lymph node sections. (A and D) Hematoxylin and eosin-stained, (B and E) immunostained, (C) negative control. C, capsule; L, lymph follicle; LV, lymph vessel; S, sinus.

The lymph nodes exhibited a weak positive result for lymphocyte aggregations in three of five cases (3/5), while one case (1/5) demonstrated a strong positive result, and one case (1/5) exhibited a negative result. The lymph node capsule, trabeculae and sinus exhibited weak positive results in all cases. In one sample, the sinus was fibrotic and could not be evaluated (Fig. 1A-C).

Additionally, in certain sections (1/5), the lymph vessels were incised, resulting in a positive staining outcome. However, due to the limited sample size, a definitive analysis could not be conducted (Fig. 1D and E).

### Spleen

**Figure 2:**
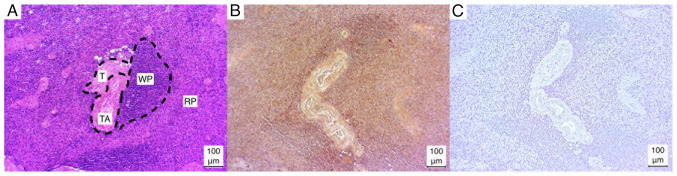
Microphotographs of spleen sections. (A) Hematoxylin and eosin-stained, (B) immunostained, (C) negative control. RP, red pulp; T, trabecula; TA, trabecula artery; WP, white pulp.

In the case of the lymphocyte aggregations in the white pulp, either a strong positive (2/5) or a weak positive (3/5) result was observed. By contrast, the red pulp exhibited a weak positive result in the majority of cases (4/5), with only one instance of a strong positive result (1/5). In addition, the capsule and trabeculae exhibited a consistently weak positive staining result in all cases (5/5). Similarly, the trabecular arteries exhibited a consistent, weak positive result, with the media of the vessels particularly susceptible to staining (Fig. 2).

### Palatine tonsil

**Figure 3:**
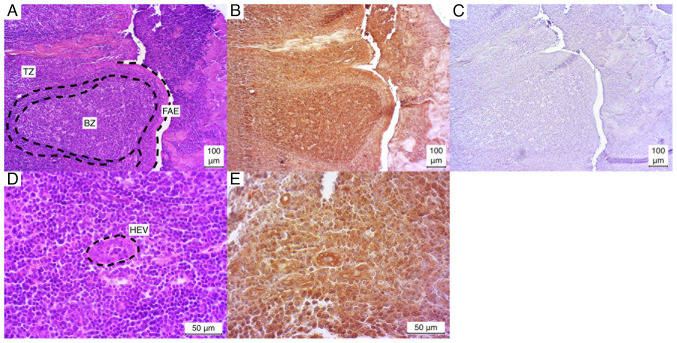
Microphotographs of palatine tonsil sections. (A and D) Hematoxylin and eosin-stained, (B and E) immunostained, (C) negative control. BZ, B-zone (lymph follicle); FAE, follicle-associated epithelium; HEV, high endothelial venule; TZ, T-zone.

The palatine tonsil exhibited a markedly positive IHC staining pattern for the observed secondary follicles in all three cases (3/3). The germinal center was particularly well-stained in comparison to the surrounding edge. In contrast, the T-zone demonstrated a relatively weak positive staining pattern in all three cases (3/3). The follicle associated epithelium (FAE) and the incised high endothelial venules (HEVs) exhibited a similarly robust positive result in all three cases (Fig. 3).

### Ileum

**Figure 4:**
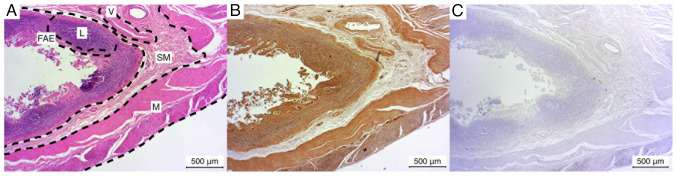
Microphotographs of ileum sections. (A) Hematoxylin and eosin-stained, (B) immunostained, (C) negative control. FAE, follicle-associated epithelium; L, lymphocyte aggregation; M, muscularis; SM, submucosa; V, vessels.

The ileum exhibited a weak positive staining result for lymphocyte accumulations in the majority of cases (3/5), while in one additional case (1/5), the lymphocytes demonstrated a negative staining result. In one instance (1/5), Peyer's patches were not observed, thus precluding the assessment of the lymphocytes and FAE. In one of five cases (1/5), the FAE exhibited a markedly positive staining result. In the remaining cases, the staining was weak positive (3/5). The submucosa and the embedded vessels and muscularis exhibited a consistently weak positive result in all five cases (Fig. 4).

### Vermiform appendix

**Figure 5:**
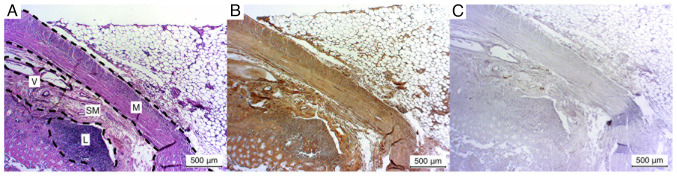
Microphotographs of vermiform appendix sections. (A) Hematoxylin and eosin-stained, (B) immunostained, (C) negative control. L, lymphocyte aggregation; M, muscularis; SM, submucosa; V, vessels.

The results of the vermiform appendix exhibited a comparable trend to those of the ileum. In the majority of cases (3/5), a weak positive staining of the lymphocyte aggregation in the Peyer's patches was observed. In the remaining cases (2/5), the lymphocyte staining was negative. The FAE exhibited a markedly positive staining result in two of the five cases (2/5), while the remaining three cases demonstrated a weak positive result (3/5). The submucosa and the embedded vessels and muscular layer exhibited a consistently weak positive staining result in all five cases (Fig. 5).

### Thymus

**Figure 6:**
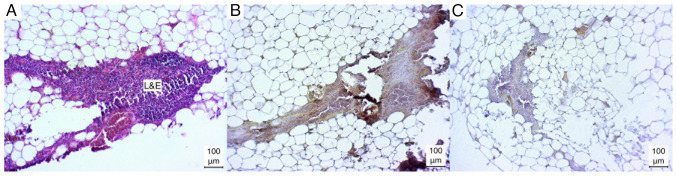
Microphotographs of thymus sections. (A) Hematoxylin and eosin-stained, (B) immunostained, (C) negative control. L&E, lymphocyte aggregation with incorporated epithelial cells.

In the majority of cases (3/5), the thymus tissue exhibited a weak positive result for lymphocyte immunostaining. Conversely, in the remaining cases (2/5), the lymphocyte immunophenotyping yielded a negative result. The intermediate epithelial cells demonstrated a weak positive immunostaining in four of the five cases (4/5). In one instance, a negative result was observed (1/5). The overall result of the thymus tissue staining was the weakest (Fig. 6).

## Discussion

The present study demonstrated the presence of TRPC6 protein in all investigated lymphatic tissues. To the best of our knowledge, this represents the first direct detection of the protein in lymphatic tissues.

The specificity of the antibody has been corroborated in previous studies that utilized the same antibody from Alomone Labs through peptide incubation procedures and knockout validation experiments (36,37). Our study established positive control samples for the same tissue type, cardiac muscle, which allowed for comparison with the previously mentioned study by Jacobs *et al* (30).

The TRPC6 protein was identified in lymphocytes in all examined tissues, with notable variations observed across different organs. In the spleen, the white pulp exhibited stronger immunostaining than the lymphocyte aggregates in lymph nodes, ileum, appendix and thymus. This pattern aligns with the previously described expression patterns of the TRPC6 gene in murine lymphatic tissues (38).

However, the strongest staining signal was observed in the lymphocytic population of the palatine tonsil. This observation may be attributed to the fact that the tissue was obtained from young patients undergoing tonsillectomy. A differential staining behavior between T- and B-lymphocytes was also observed, with the B-zone exhibiting a darker staining pattern than the T-zone in the tonsil. The results are consistent with the previously described TRPC6 expression patterns in murine T- and B-lymphocytes. In this case, the B-lymphocytes exhibited a significantly stronger expression than the T-lymphocytes (33).

The TRPC6 protein was identified in follicle-associated lymphatic tissue in both the tonsil and gut-associated lymphatic tissue. TRPC6 may be involved in molecular processes of the immune response.

The lymph vessels in lymph nodes, the vessels in the submucosa of the vermiform appendix and ileum, as well as the trabecular arteries of the spleen expressed TRPC6. This was similarly described for vessels in non-lymphatic organs in the study by Abdinghoff *et al* (39). The expression of the TRPC6 protein in murine lymph vessels has already been described (40). The intima and adventitia exhibited particularly robust staining in the IHC analysis. It is noteworthy that the lymphatic vessels were only incised in one of the observed sections, limiting the ability to draw conclusions about TRPC6 expression lymphatic vessels.

The observation of HEVs in the palatine tonsil demonstrated a notable presence of TRPC6 protein. As TRPC6 functions as a mediator in leukocyte migration (28), it was hypothesized that this process in HEVs may be associated with TRPC6. This assertion is limited to granulocytes at present, however. A connection between TRPC6 and the diapedesis of lymphocytes through the HEV has not been described and cannot be proven by the methodology of the present study. Unfortunately, HEV could not be observed in the other examined tissues, which can be attributed to processes of immunosenescence (41).

The most notable limitation of the present study was the advanced age of the donors, with an average age of 83 years at death. Consequently, the evidence presented herein is limited to the lymphatic tissue of the elderly, with the exception of the tonsils. As only tonsillar tissue from young subjects was examined, it cannot be ruled out that the comparability of the results to the other examined tissues may be affected by the age difference. It was possible to identify age-related morphological changes in the lymph nodes and thymus, as well as in the Peyer's patches in the ileum and vermiform appendix. The thymus tissue exhibited the presence of lymphocytic aggregates in the form of thymal residual tissue networks within the retrosternal adipose tissue. Hassall's corpuscles could not be observed. These findings were corroborated upon repeated tissue sampling. The observed morphology of the thymus tissue aligns with the previously described age-related changes in morphology in the study by Ströbel *et al* (42). In the lymph nodes, there was a shift in the boundary between the cortex and the medulla, a fibrotic transformation, a reduction in the number of HEVs and secondary follicles (41,43-45). Consequently, it was not possible to differentiate between the T- and B-zones in the tissues, apart from the tonsilla, as these zones merge during the immunosenescence (44,46).

The consistent result of the immunostaining of the connecting tissues in lymphatic organs can be linked to the discovery that TRPC6 plays a central role in the differentiation of fibroblasts (47). This suggests a potential link between TRPC6 and the development of fibrosis.

The utilization of IHC immunostaining to detect TRPC6 in human lymphatic tissues represents a limitation of the present study. Further assays, such as western blotting or RT-PCR, need to be performed to substantiate the evidence that TRPC6 is ubiquitously present in human lymphatic tissues.

Furthermore, the morphology and antigen preservation are also affected by autolytic processes that arise during the time periods immediately preceding the fixation process (48). It is not possible to exclude the possibility of autolytic processes occurring as a result of the methodology employed in this study. Moreover, it cannot be ruled out that the applied fixation method influenced the antigen preservation, despite the absence of differences in staining behavior between fixed specimens of the same organ, where dissimilar fixation techniques were employed.

In conclusion, research on TRPC6 in lymphatic tissue is still in its infancy. Nevertheless, the present study indicates that the widespread presence of TRPC6 in this tissue suggests a diverse potential range of functions of the TRPC6 channel in lymphatic tissue. The identification of drugs that interact with TRPC6 (21,49) could open new avenues for therapeutic intervention in autoimmune diseases, septic syndromes and malignant lymphatic diseases. Potential treatments could target TRPC6 to control the release of cytokines in sepsis and inhibit abnormal calcium signaling in cancers such as B-cell lymphomas. The results of the present study emphasize the promising future applications of pharmacological interventions in these diseases. However, the ubiquity of TRPC6 in lymphatic tissue demonstrated in our study is likely to pose a major challenge to the selectivity of any potential therapy. Further IHC studies of tissue from young subjects, as well as pathological tissue, are necessary. Of particular interest would be an immunohistochemical study of TRPC6 in lymphoma cells or the staining of lymph nodes of septic patients, with a direct comparison to physiological tissue. This could provide deeper insight into the role of TRPC6 in pathophysiological functions in lymphatic tissue and could aid the development of pharmaceutical therapies.

## Figures and Tables

**Table I:** Collected samples overall.

Sample origin	First collection	Second collection	Overall
Lymph nodes	10	-	10
Spleen	13	-	13
Palatine tonsil	3	-	3
Ileum	10	10	20
Vermiform appendix	4	8	12
Thymus	10	34	44

**Table II:** Information of corresponding body donors of the evaluated immunostained samples.

A, Body donors of lymph node samples								
Sample origin	Fixation	Sex	Age, years	Cause of death	Lymphocyte aggregations	Lymph node sinus, capsule and trabeculae		Lymph node vessels
Lymph nodes	NEP	F	81	Cardiac arrest	-	+		N.e.
	NEP	F	87	Multiorgan failure	++	N.e.		N.e.
	Formalin	F	83	Cerebral herniation	+	+		+
	Formalin	M	98	Cardiac arrest	+	+		N.e.
	NEP	M	85	Cardio-pulmonary failure	+	+		N,e.
B, Body donors of spleen samples								
Sample origin	Fixation	Sex	Age, years	Cause of death	White pulp	Red pulp	Capsule and trabeculae	Trabecular arteries
Spleen	NEP	F	87	Multiorgan failure	++	+	+	+
	NEP	F	79	Multiorgan failure	++	++	+	+
	NEP	F	85	Cardio-pulmonary failure	+	+	+	+
	NEP	F	90	Consumption coagulopathy	+	+	+	+
	NEP	F	81	Cardiac arrest	+	+	+	+
C, Body donors of tonsil samples								
Sample origin	Fixation	Sex	Age, years	Cause of death	Secondary follicle	T-zone	Follicle-associated epithelium	High endothelial venule
Palatine tonsil	Formalin (D.i.)	M	47	-	++	+	++	++
	Formalin (D.i.)	F	18	-	++	+	++	++
	Formalin (D.i.)	M	32	-	++	+	++	++
D, Body donors of ileum samples								
Sample origin	Fixation	Sex	Age, years	Cause of death	Lymphocyte accumulations	Follicle associated epithelium	Submucosa and embedded vessels	Muscularis
Ileum	NEP	F	90	Consumption coagulopathy	++	+	+	+
	NEP	F	87	Multiorgan failure	N.e.	N.e.	+	+
	Formalin	M	72	Cerebral hypoxia	+	-	+	+
	NEP	F	75	Advanced anal carcinoma	+	++	+	+
	Formalin	F	84	Multiorgan failure	+	+	+	+
E, Body donors of appendix samples								
Sample origin	Fixation	Sex	Age, years	Cause of death	Lymphocyte accumulations	Follicle-associated epithelium	Submucosa and embedded vessels	Muscularis
Vermiform	Formalin	M	72	Cerebral hypoxia	++	+	+	+
appendix	Formalin	M	81	Pneumonia	+	-	+	+
	NEP	F	75	Advanced anal carcinoma	++	+	+	+
	Formalin	M	92	Multiorgan failure	+	-	+	+
	Formalin	F	82	Age-related mortality	+	+	+	+
F, Body donors of thymus samples								
Sample origin	Fixation	Sex	Age, years	Cause of death	Lymphocyte aggregations		Epithelial cells	
Thymus	Formalin	F	84	Multiorgan failure	+		+	
	Formalin	F	84	Multiorgan failure	+		+	
	Formalin	M	80	Circulatory failure	-		-	
	Formalin	F	89	Cardiac arrest	+		+	
	Formalin	M	92	Multiorgan failure	-		+	

D.i, Directly immersed; F, female; M, male; NEP, nitric pickling salt and ethanol; N.e., Not evaluable; -, negative, +, weak positive; ++, strong positive.

## Data Availability

The datasets used and/or analyzed during the current study are available from the corresponding author on reasonable request.
